# Cerebral Catheter Angiography Findings in Adult Filipinos: A Quantitative Study of Intracranial Vessel Diameters and Flow Patterns

**DOI:** 10.7759/cureus.88490

**Published:** 2025-07-22

**Authors:** Miguel Nicolai T Victorino, Reynaldo Benedict V Villamor, Anna Marie L Bernardo, Lester Ron S Bustamante, Jaime Lois III F Opinion

**Affiliations:** 1 Neurosurgery, Vicente Sotto Memorial Medical Center, Cebu City, PHL

**Keywords:** cerebral catheter angiography, filipino, intracranial vessels, neuroimaging, vascular anatomy

## Abstract

Introduction

Cerebral catheter angiography is crucial for cerebrovascular disorder diagnosis, but local normative data on intracranial vessel characteristics in healthy Filipino adults are lacking. Individual variations in Circle of Willis components highlight the need for establishing Filipino-specific vessel size norms to aid diagnosis and neurosurgical planning.

Methods

This retrospective study analyzed 73 cerebral catheter angiograms from 1099 patients at Vicente Sotto Memorial Medical Center (2015-2021). Patients with known vascular pathologies or under 18 were excluded; 222 angiograms were further excluded for atherosclerosis/vasospasm. Mean, standard deviation, and 95% confidence intervals were computed for vessel diameters. Normality (Kolmogorov-Smirnov, Shapiro-Wilk) and laterality differences (Levene's, t-tests) were assessed. Anterior cerebral artery (ACA) flow patterns and transverse sinus dominance were reported as percentages. All analyses used IBM SPSS Statistics for Windows (IBM Corp., Version 22.0, Armonk, NY).

Results

Normal intracranial vessel diameter ranges (mm) were as follows: internal carotid artery (ICA) supraclinoid (3.37-3.74), middle cerebral artery (MCA) M1 (2.54-2.74), MCA M2 superior (1.83-1.99), MCA M2 inferior (1.79-1.96), posterior cerebral artery (PCA) (1.82-2.00), superior cerebellar artery (SCA) (1.22-1.34), posterior inferior cerebellar artery (PICA) (1.46-1.66), vertebral artery (2.99-3.38), basilar artery (3.14-3.45). Significant laterality differences (mm) were found for ACA A1: right (1.87-2.01), left (1.93-2.20); posterior communicating artery (PCOM): right (1.69-1.78), left (1.57-1.98); and transverse sinus: right (6.68-7.90), left (5.14-6.11). Anterior cerebral flow was 53.4% symmetrical (23.3% right-dominant, 23.3% left-dominant). Right transverse sinus dominance was 56.2%, symmetrical inn 28.8%, and left-dominant in 15.1%. Fetal PCOM was 29%.

Conclusion

This study presents the first comprehensive normal intracranial vessel sizes for adult Filipinos. Filipino vessel diameters are notably smaller than international reports, likely due to ethnicity and measurement methodology. These data serve as a vital reference for managing Filipino patients where vessel size dictates therapeutic, prognostic, and diagnostic decisions. Observed arterial flow patterns and sinus dominance also differ from Western studies, offering further aid in Filipino patient care.

## Introduction

The rapidly advancing field of neuroendovascular treatments has increased reliance on cerebral catheterization to evaluate intracranial arteries. Although studies show demographic differences in adult brains, a thorough examination of population and related factors affecting brain structure and microstructure has not yet been conducted. Establishing normative data from diverse populations, without bias toward a single group, is crucial for objectively studying normal and abnormal brain development. Few studies compare intracranial vessel differences across various healthy populations. Variations in the length and diameter of arteries forming the Circle of Willis have been observed [1]. While many studies have detailed the anatomy of individual segments of the cerebral arterial circle, only a few have examined the variations of the entire circle [2]. Researchers have identified anomalies affecting vessel diameters, yet the normal ranges for these vessels remain unreported. Moreover, there has been no comparison of how often these variants occur among different ethnic or racial groups.

Cerebral angiography, developed after pneumoencephalography, remains a crucial imaging tool for assessing cerebrovascular function. Over the last two decades, non-invasive brain vascular imaging methods, such as computed tomography angiography (CTA) and magnetic resonance angiography (MRA), have become increasingly accessible, widely used, and effective [3]. Despite progress in spatial resolution, acquisition speed, and 3D post-processing capabilities, the role of the more invasive and costly catheter cerebral angiogram is still debated, with its importance possibly decreasing. Although CT angiography is generally safe, it involves catheterization of the carotid and vertebral arteries, posing a small risk of severe neurological complications [4]. The first intracranial vessel catheterization using a Silastic microcatheter, performed by Luessenhop and Velasquez, marked a significant milestone in the field. This technique has since become the preferred imaging method for many vascular neurosurgical conditions [5]. Digital subtraction angiography (DSA) is increasingly favored for diagnosing and treating various neurovascular disorders [6]. Traditional angiography involves X-ray imaging of the target area with contrast injected via microcatheters [7]. At the same time, DSA captures a baseline image and subtracts subsequent images in real-time for comprehensive monitoring [6]. Studies have shown that DSA is superior to multidetector computed tomography (MDCT) angiography in detecting small intracranial arteries, as it can visualize vessels as small as 0.4 mm, compared to MDCT's detection limit of 0.7 mm [8].

Previous research efforts aimed to establish normative vascular data. A study established criteria for the standard size and position of the basilar artery (BA) to aid in diagnosing conditions such as vertebrobasilar dolichoectasia [9]. Cadaver studies dissected specimens to measure vessel lengths and diameters of the Circle of Willis, reporting mean internal cerebral artery diameters of 4.2 ± 0.9 mm, anterior cerebral arteries of 2.2 ± 0.6 mm (right) and 2.4 ± 0.5 mm (left) [10], and posterior cerebral arteries of 2.11 ± 0.7 mm (right) and 2.2 ± 0.6 mm (left) [2]. Perlmutter and Rhoton’s cadaveric study indicated an average diameter of 2.6 mm for both the left and right anterior cerebral arteries [10]. Another study using DSA found the mean diameter of the first segment of the middle cerebral artery (MCA) to be 3.26 ± 0.077 mm, and the second segment to be 2.62 ± 0.5 mm [11]. High-resolution CT studies reported a mean BA diameter of 3.17 mm, with a range from 1.86 to 4.53 mm [9]. Researchers examined the internal cerebral artery, ACA A1 segment, MCA M1 and M2 segments, vertebral artery, BA, and PCA P1 segment as potential sites for critical strokes [8]. Cranial venous sinus dominance was also studied, revealing 36% right transverse sinus dominance, 14% left, and 50% bilateral dominance, defined by a right-to-left measurement ratio greater than 1.5 or less than 0.67 [12]. A study noted that this is a promising noninvasive alternative to the current standard intra-arterial digital subtraction angiography (IA-DSA) [4]. International data suggest average diameters such as BA, 3.4 ± 0.8 mm; right vertebral artery (RVA), 2.2 ± 0.8 mm; left vertebral artery (LVA), 2.8 ± 0.8 mm; right internal carotid artery (RICA), 4.7 ± 0.7 mm; left internal carotid artery (LICA), 4.7 ± 0.8 mm; right middle cerebral artery (RMCA), 2.4 ± 0.4 mm; and left middle cerebral artery (LMCA), 2.4 ± 0.5 mm [13]. Notably, none of these measurements were obtained using Filipino patient data.

Understanding the normal size of these vessels in the local context is crucial for neurosurgeons to enhance diagnosis and evaluate surgical options for patients. Most existing data on intracranial vessel size originates from Western countries with larger populations and more extensive academic resources. For the Philippines, such data are unavailable. Although this study does not intend to do a comparative analysis, it aims to fill that gap by establishing a normative baseline of intracranial arterial and venous dimensions and flow patterns specifically for adult Filipinos, considering that the study’s local population has the highest volume of cerebral angiography findings in the whole Visayas region in the Philippines.

General objective

This study’s general objective is to enable a quantitative survey of arterial diameters in Filipinos, aiding more accurate assessment of cerebral vasospasm, clinically significant stroke, and other conditions that require precise measurements of Filipino arteries.

Specific Objectives

Specifically, this study aimed to determine the mean diameter of both the left and right sides of the following intracranial vessels: internal carotid artery (ICA) - supraclinoid, anterior cerebral artery (ACA) - first segment, MCA - first segment, MCA - second segment superior trunk, MCA - second segment inferior trunk, vertebral artery - V4 segment, BA, posterior cerebral artery (PCA) - first segment, superior cerebellar artery (SCA), posterior inferior cerebellar artery (PICA), posterior communicating artery (PCOM), and anterior communicating artery; determine the proportion of filling of the anterior cerebral circulation in terms of ACA: right dominant flow type, left dominant flow type, and symmetrical flow type; determine the dominant transverse sinus: right dominant transverse sinus, left dominant transverse sinus, and symmetrical transverse sinus; and determine the presence of fetal type PCOM, categorized as either fetal type PCOM or non-fetal type PCOM.

## Materials and methods

Study design

This study employed a retrospective cross-sectional design, collecting data on all variables over a defined period without conducting follow-up assessments. It determined the average diameter of major intracranial blood vessels, identified patterns of cerebral artery flow, the incidence of fetal-type posterior communicating arteries, and assessed the dominance of the transverse sinus among adult Filipinos admitted to Vicente Sotto Memorial Medical Center (VSMMC).

Study setting

The study was carried out at VSMMC in Cebu City, Central Visayas, Philippines. VSMMC is the largest government hospital in the Visayas, with nearly 1,500 beds, serving as the main referral hospital for the Visayas region and upper Mindanao. Specifically, the study took place in the VSMMC Department of Neurosurgery, which performs over 1000 brain and spine surgeries each year.

Population and sampling technique

The population included all patients admitted to VSMMC from January 2015 to December 2021. Patients were chosen based on the inclusion and exclusion criteria established for the study. The study encompassed all adult patients who underwent cerebral catheter angiography.

Inclusion Criteria

This study included all adult patients admitted to VSMMC who underwent cerebral catheter angiography from January 2015 to December 2021.

Exclusion Criteria

Patients with dural arteriovenous fistulas, arteriovenous malformations, dissecting aneurysms, and lesions causing displacement of blood vessels, such as hydrocephalus, brain tumors, and hematomas, as well as those demonstrating radiologic features of vasospasm and atherosclerosis and all other vascular pathology that could influence or change the caliber of the blood vessels such as Moyamoya disease, are excluded from the study.

Data processing and analysis

The cerebral angiograms of patients included in the study were collected and coded. These were then randomly assigned to the researchers, and the anatomical measurements were performed using Horos Reader (Horos Project, Annapolis, MD). Patients with incomplete measurements due to difficulty visualizing the vessel structures were excluded from the study. Descriptive statistics summarized the characteristics and measurements. Frequency and proportion were used for laterality and patterns, while means and standard deviations were used for diameters. Confidence intervals at the 95% level were also reported. The Shapiro-Wilk test was used to assess the normality of the diameter measurements. Group means based on laterality (right versus left or fetal versus non-fetal) were calculated and compared using an independent sample t-test. The equality of variances was tested with Levene’s test. A p-value of 0.05 or less was considered statistically significant. All calculations and tests were performed using IBM SPSS for Windows (IBM Corp., Version 22.0, Armonk, NY).

Scope and limitations

The scope of this study includes postoperative patients who underwent cerebral catheter angiography at the VSMMC Department of Neurosurgery. It is limited to eligible patients from the VSMMC from January 2015 to December 2021. The scope was also restricted due to a limited set of variables, which was caused by time constraints. Additionally, the retrospective nature of this study depended solely on the attending physicians' compliance in recording detailed patient information. Furthermore, data validation was not possible because it relied on the availability of the physicians or staff who admitted and treated the patients. The accuracy and reliability of the data collected depend on the correctness of patient records and angiogram results. To reduce errors caused by these limitations, complete enumeration can help ensure that discrepancies from their actual parameters are minimized. Similarly, data cleaning ensures the completeness of data for accurate analysis.

Ethical considerations

Primarily, this investigation is guided by ethical considerations for conducting chart reviews. The investigator sought approval from the VSMMC’s Research Institute (VRI) and Research Ethics Committee (VSMMC-REC) to carry out the study. Data collection was only performed after approval was obtained. According to the Data Privacy Act of 2012, the confidentiality and anonymity of participants were protected, with coding maintained throughout the study. All participants were de-identified and assigned unique alphanumeric codes. The investigators maintained the link between these codes and did not disclose it elsewhere. A data extraction tool was used to record relevant data from patients’ charts, and the information was extracted and encoded using Google Sheets (Google, Inc., Mountain View, CA). Upon completion, hard copies were submitted to the research committee and the Department of Neurosurgery office, in accordance with the training program's documentation requirements. The principal investigator will destroy all data stored in Google Sheets. Before termination, all data will be exported to a password-protected physical hard drive and stored securely in a locked cabinet for a period of 10 years. After 10 years, the data on the hard drive will be destroyed by the researchers. Access to the collected data will be restricted solely to the investigators of this study. The researcher also ensured that no patient names or personal information were included in the final report. The researchers guarantee that only they have access to the data, which was used exclusively for this study. This investigation ensures that the benefits outweigh the risks, as it only involves a chart review with no direct contact with live human subjects, especially during the current pandemic. No funding was sought for this research; all expenses were borne by the researchers. The researchers also declare no conflict of interest in conducting this study.

## Results

Table 1 presents the average ICA diameter. The average for all 146 patients was 3.63 mm (SD = 0.76, 95% CI: 3.50, 3.75). The average ICA diameter by side was also calculated: for the right side, it was 3.55 mm (SD = 0.77, 95% CI: 3.37-3.74), and for the left side, 3.70 mm (SD = 0.74, 95% CI: 3.53-3.87). The results showed no significant difference in mean diameter (t(144) = -1.165, p = 0.246).

**Table 1:** Internal Cerebral Artery Supraclinoid Segment

Mean (SD), mm 95% CI			Mean diff (SE), mm	t(df)	p-value
All (n = 146)	Right (n =73)	Left (n = 73)	95% CI		
3.63 (0.76) 3.50 to 3.75	3.55 (0.77) 3.37 to 3.74	3.70 (0.74) 3.53 to 3.87	-0.14 (0.13) -0.39 to 0.14	t(144) = -1.165	0.246

Table 2 presents the first segment of the ACA (A1), with a mean diameter of 1.98 mm (SD = 0.54, 95% CI: 1.89-2.07) in 143 patients. The mean A1 diameter was also calculated by side: on the right, it was 1.89 mm (SD = 0.48, 95% CI: 1.78-2.01), and on the left, it was 2.07 mm (SD = 0.58, 95% CI: 1.93-2.20). Results indicated a significant difference of 0.18 mm between sides (t(141) = -1.980, p = 0.050).

**Table 2:** Anterior Cerebral Artery - First Segment

Mean (SD), mm 95% CI			Mean diff (SE), mm	t(df)	p-value
All (n = 143)	Right (n = 70)	Left (n = 73)	95% CI		
1.98 (0.54) 1.89 to 2.07	1.89 (0.48) 1.78 to 2.01	2.07 (0.58) 1.93 to 2.20	-0.18 (0.09) -0.35 to -0.00	t(141) = -1.980	0.050

Table 3 shows the anterior cerebral flow pattern, with symmetrical flow being the most common. Specifically, 39 (53.4%) patients had symmetrical flow, while 17 (23.3%) patients showed left-dominant flow, and 17 (23.3%) patients exhibited right-dominant flow patterns.

**Table 3:** Anterior Cerebral Artery Flow Pattern

Patterns	Frequency (%)
Right	17 (23.3)
Left	17 (23.3)
Symmetrical	39 (53.4)

Table 4 presents the mean diameter of the first segment of the MCA (M1) in 146 patients, which was 2.64 mm (SD = 0.59, 95% CI: 2.54, 2.74). The mean M1 diameter by side was calculated as follows: 2.63 mm (SD = 0.59, 95% CI: 2.49-2.77) for the right side and 2.66 mm (SD = 0.59, 95% CI: 2.52-2.78) for the left side. The comparison revealed no significant difference in mean diameter (t(144) = -0.304, p = 0.762).

**Table 4:** Middle Cerebral Artery - First Segment

Mean (SD), mm 95% CI			Mean diff (SE), mm	t(df)	p-value
All (n = 146)	Right (n = 73)	Left (n = 73)	95% CI		
2.64 (0.59) 2.54 to 2.74	2.63 (0.59) 2.49 to 2.77	2.66 (0.59) 2.52 to 2.78	-0.03 (0.10) -0.22 to 0.16	t(144) = -0.304	0.762

Table 5 presents the MCA superior trunk with the average diameter of 1.91 mm (SD = 0.49, 95% CI: 1.83-1.99) in 146 patients. The mean MCA superior diameter based on laterality was calculated as follows: for the right side, the average was 1.93 mm (SD = 0.50, 95% CI: 1.81-2.05), and for the left side, it was 1.89 mm (SD = 0.49, 95% CI: 1.77-2.00). The mean comparison showed no significant difference in diameter (t(144) = 0.542, p = 0.589).

**Table 5:** Middle Cerebral Artery - Second Segment Superior Trunk

Mean (SD), mm 95% CI			Mean diff (SE), mm	t(df)	p-value
All (n = 146)	Right (n = 73)	Left (n = 73)	95% CI		
1.91 (0.49) 1.83 to 1.99	1.93 (0.50) 1.81 to 2.05	1.89 (0.49) 1.77 to 2.00	0.04 (0.08) -0.12 to 0.21	t(144) = 0.542	0.589

Table 6 presents the inferior trunk of the MCA with the mean diameter of 1.87 mm (SD = 0.53, 95% CI: 1.79, 1.96) in 146 patients. The mean diameter of the MCA inferior trunk based on laterality was calculated as follows: for the right side, the mean diameter was 1.89 mm (SD = 0.52, 95% CI: 1.77, 2.01), while for the left side, it was 1.85 mm (SD = 0.55, 95% CI: 1.73, 1.98). The comparison of means showed no significant difference in diameter (t(144) = 0.410, p = 0.683).

**Table 6:** Middle Cerebral Artery - Second Segment Inferior Trunk

Mean (SD), mm 95% CI			Mean diff (SE), mm	t(df)	p-value
All (n = 146)	Right (n = 73)	Left (n = 73)	95% CI		
1.87 (0.53) 1.79 to 1.96	1.89 (0.52) 1.77 to 2.01	1.85 (0.55) 1.73 to 1.98	0.04 (0.09) -0.14 to 0.21	t(144) = 0.410	0.683

Table 7 presents the mean diameter of the PCOM among 65 patients, which was 1.74 mm (SD = 0.55, 95% CI: 1.60-1.87). By side, the mean diameter for the right was 1.69 mm (SD = 0.53, 95% CI: 1.51-1.88), and for the left, it was 1.78 mm (SD = 0.58, 95% CI: 1.57-1.98). The difference in mean diameter was not significant (t(63) = -0.599, p = 0.551).

**Table 7:** Posterior Communicating Artery

Mean (SD), mm 95% CI			Mean diff (SE), mm	t(df)	p-value
All (n = 65)	Right (n = 33)	Left (n = 32)	95% CI		
1.74 (0.55) 1.60 to 1.87	1.69 (0.53) 1.51 to 1.88	1.78 (0.58) 1.57 to 1.98	-0.08 (0.14) -0.36 to 0.19	t(63) = -0.599	0.551

Table 8 shows the comparison conducted between the fetal and non-fetal groups. The fetal group had a mean diameter of 2.03 mm (SD = 0.31, 95% CI: 1.88-2.17), while the non-fetal group’s mean was 1.61 mm (SD = 0.58, 95% CI: 1.44-1.79). This showed a significant difference of 0.41 mm (t(63) = -2.904, p = 0.005). Nineteen out of 65 patients (29%) exhibited a fetal type of PCOM.

**Table 8:** Posterior Communicating Artery - Fetal Flow Type

Mean (SD), mm 95% CI		Mean diff (SE), mm	t(df)	p-value
Fetal (n = 19)	Non-fetal (n = 46)	95% CI		
2.03 (0.31) 1.88 to 2.17	1.61 (0.58) 1.44 to 1.79	-0.41 (0.14) -0.69 to -0.13	t(63) = -2.904	0.005

Table 9 presents the transverse sinus, with which 136 patients had a mean diameter of 6.49 mm (SD = 2.44, 95% CI: 6.08, 6.91). The mean transverse sinus diameter based on laterality was calculated as follows: on the right side, the mean diameter was 7.29 mm (SD = 2.57, 95% CI: 6.68, 7.90), while on the left side, it was 5.62 mm (SD = 1.96, 95% CI: 5.14, 6.11). Results demonstrated a significant difference in mean diameter of 1.66 mm between sides (t(134) = 4.221, p < 0.001).

**Table 9:** Transverse Sinus

Mean (SD), mm 95% CI			Mean diff (SE), mm	t(df)	p-value
All (n = 136)	Right (n = 71)	Left (n = 65)	95% CI		
6.49 (2.44) 6.08 to 6.91	7.29 (2.57) 6.68 to 7.90	5.62 (1.96) 5.14 to 6.11	1.66 (0.39) 0.88 to 2.44	t(134) = 4.221	<0.001

Table 10 presents the distribution of transverse sinus dominance, with the majority exhibiting right-sided dominance (56.2%), followed by symmetrical dominance (28.8%). Left-sided dominance was the least common, observed in only 15.1% of patients. 

**Table 10:** Transverse Sinus Dominance

Dominance	Frequency (%)
Right	41 (56.2)
Left	11 (15.1)
Symmetrical	21 (28.8)

Table 11 presents the PCA with the mean diameter of 1.91 mm (SD = 0.52, 95% CI: 1.82-2.00) in 134 patients. The mean PCA diameter based on laterality was also calculated as follows: for the right side, the mean diameter was 1.96 mm (SD = 0.51, 95% CI: 1.83-2.08), while for the left side, it was 1.86 mm (SD = 0.53, 95% CI: 1.74-1.99). An average comparison showed no significant difference in mean diameter (t(132) = 1.034, p = 0.303).

**Table 11:** Posterior Cerebral Artery - First Segment

Mean (SD), mm 95% CI			Mean diff (SE), mm	t(df)	p-value
All (n = 134)	Right (n = 67)	Left (n = 67)	95% CI		
1.91 (0.52) 1.82 to 2.00	1.96 (0.51) 1.83 to 2.08	1.86 (0.53) 1.74 to 1.99	0.09 (0.09) -0.08 to 0.27	t(132) = 1.034	0.303

Table 12 presents the SCA with the mean diameter of 1.28 mm (SD = 0.36, 95% CI: 1.22-1.34) in 136 patients. The mean SCA diameter based on laterality was also calculated as follows: for the right side, the mean diameter was 1.27 mm (SD = 0.35, 95% CI: 1.18-1.35), while for the left side, it was 1.29 mm (SD = 0.38, 95% CI: 1.20-1.38). A mean comparison showed no significant difference in diameter (t(134) = -0.408, p = 0.684).

**Table 12:** Superior Cerebellar Artery

Mean (SD), mm 95% CI			Mean diff (SE), mm	t(df)	p-value
All (n = 136)	Right (n = 68)	Left (n = 68)	95% CI		
1.28 (0.36) 1.22 to 1.34	1.27 (0.35) 1.18 to 1.35	1.29 (0.38) 1.20 to 1.38	0.09 (0.09) -0.08 to 0.27	t(134) = -0.408	0.684

Table 13 presents the mean of PICA diameter in 66 patients, which was 1.56 mm (SD = 0.41, 95% CI: 1.46, 1.66). The mean PICA diameter by side was also calculated: for the right side, it was 1.61 mm (SD = 0.45, 95% CI: 1.46-1.75), and for the left side, it was 1.48 mm (SD = 0.34, 95% CI: 1.35-1.62). Comparison indicated no significant difference in mean diameter (t(64) = -1.190, p = 0.238).

**Table 13:** Posterior Inferior Cerebellar Artery

Mean (SD), mm 95% CI			Mean diff (SE), mm	t(df)	p-value
All (n = 66)	Right (n = 39)	Left (n = 27)	95% CI		
1.56 (0.41) 1.46 to 1.66	1.61 (0.45) 1.46 to 1.75	1.48 (0.34) 1.35 to 1.62	-0.12 (0.10) -0.33 to 0.08	t(64) = -1.190	0.238

Table 14 presents the vertebral artery with the mean diameter of 3.18 mm (SD = 0.82, 95% CI: 2.99-3.38) in 69 patients. The mean vertebral diameter based on laterality was also calculated as follows: for the right side, the mean diameter was 3.09 mm (SD = 0.85, 95% CI: 2.68-3.50), while for the left side, it was 3.22 mm (SD = 0.82, 95% CI: 2.99-3.45). The mean comparison revealed no significant difference in diameter (t(67) = -0.581, p = 0.563).

**Table 14:** Vertebral Artery

Mean (SD), mm 95% CI			Mean diff (SE), mm	t(df)	p-value
All (n = 69)	Right (n = 19)	Left (n = 50)	95% CI		
3.18 (0.82) 2.99 to 3.38	3.09 (0.85) 2.68 to 3.50	3.22 (0.82) 2.99 to 3.45	0.13 (0.22) -0.31 to 0.57	t(67) = -0.581	0.563

## Discussion

The cerebral catheter angiography method used to measure diameter is superior to cadaveric, CTA, or MRA because it provides real-time cine imaging of the patient's intracranial vasculature and accurate measurement of the true lumen size. However, cerebral catheter angiography is more invasive, and studies show that patients have less than a 1% risk of adverse effects such as post-puncture site hematoma, stroke, or ruptured blood vessels [14,15].

Based on the results collected, the blood vessel diameters of the ICA, MCA first segment, MCA second superior and inferior segments, PCOM, PCA, PICA, SCA, and vertebral artery show no significant differences between their sides, so that a standard mean diameter can be used. However, the ACA first segment and the transverse sinus demonstrate statistically significant differences in laterality. The initial aim of the study was to measure the left and right intracranial vessels bilaterally in all 73 patients, resulting in a total of 146 measurements. However, this was not achievable as some bilateral injections were not performed. Additionally, some participants were excluded due to the small vessel diameter of the anterior inferior cerebellar artery observed in their angiograms.

To date, no comprehensive study in the Philippines has described the normal size of intracranial blood vessels in adult Filipinos. The differences in blood vessel diameters observed in international studies are significant compared to those of Filipinos. For example, global data suggest mean diameters for the BA of 3.4 ± 0.8 mm, the right ICA of 4.7 ± 0.7 mm, and the right MCA of 2.4 ± 0.4 mm [14], which are generally larger than our findings. Overall, Filipinos seem to have smaller blood vessels. This may be due to differences in ethnicity and measurement methods. However, this study does not aim to compare Western and Filipino populations. Instead, it serves as a reference guide for treating Filipino patients, where vessel size is crucial for management, prognosis, and diagnosis. Additionally, the flow patterns of the ACA, PCOM, and fetal origin, as well as transverse sinus dominance, exhibit some differences compared to Western studies. For instance, a survey on cranial venous sinus dominance reported 36% transverse sinus dominance, 14% left-sided, and 50% symmetrical [12], which contrasts with our findings of 56.2% right-sided, 15.1% left-sided, and 28.8% symmetrical dominance. These flow patterns could also aid in diagnosing or treating patients from the Philippines.

The use of cerebral catheterization to assess the intracranial arteries in patients is becoming more common. Understanding the normal size of these vessels in our local setting may help neurosurgeons in diagnosis and in deciding the feasibility of neurosurgical procedures for selected patients. For future research, the findings of this study can serve as a basis for further studies. Additionally, the researchers recommend conducting a multi-center survey to include a larger population.

## Conclusions

This study addresses a critical void in the literature by providing the first comprehensive description of normal intracranial blood vessel sizes in adult Filipinos. As shown in this study, Filipinos possess comparatively smaller intracranial blood vessels, as confirmed by other international studies. However, this study does not aim to compare the sizes of the Western versus the Filipino populations. Instead, this could serve as a reference guide for treating Filipino patients, where the size of the blood vessel is crucial in determining management options, prognosis, and diagnosis. The observed patterns of ACA flow, fetal PCOM origin, and transverse sinus dominance also exhibit differences from those observed in Western studies. They could similarly aid in the diagnosis and treatment of Filipino patients.
